# Vertigo in Children and Adolescents: Characteristics and Outcome

**DOI:** 10.1100/2012/109624

**Published:** 2012-01-03

**Authors:** Maayan Gruber, Raanan Cohen-Kerem, Margalit Kaminer, Avi Shupak

**Affiliations:** ^1^Department of Otolaryngology, Head and Neck Surgery, Carmel Medical Center, Haifa 34362, Israel; ^2^The Bruce Rappaport Faculty of Medicine, Israel Institute of Technology (Technion), Haifa 32000, Israel; ^3^Unit of Otoneurology, Lin Medical Center, Haifa 35152, Israel

## Abstract

*Objectives*. To describe the characteristics and outcome of vertigo in a pediatric population. *Patients*. All children and adolescents presenting with vertigo to a tertiary otoneurology clinic between the years 2003–2010 were included in the study. *Results*. Thirty-seven patients with a mean age of 14 years were evaluated. The most common etiology was migraine-associated vertigo (MAV) followed by acute labyrinthitis/neuritis and psychogenic dizziness. Ten patients (27%) had pathological findings on the otoneurological examination. Abnormal findings were documented in sixteen of the twenty-three (70%) completed electronystagmography evaluations. Twenty patients (54%) were referred to treatment by other disciplines than otology/otoneurology. A follow-up questionnaire was filled by twenty six (70%) of the study participants. While all patients diagnosed with MAV had continuous symptoms, most other patients had complete resolution. *Conclusions*. Various etiologies of vertigo may present with similar symptoms and signs in the pediatric patient. Yet, variable clinical courses should be anticipated, depending on the specific etiology. This is the reason why treatment and follow up should be specifically tailored for each case according to the diagnosis. Close collaboration with other medical disciplines is often required to reach the correct diagnosis and treatment while avoiding unnecessary laboratory examinations.

## 1. Introduction

Vertigo is an uncommon complaint in children and adolescents. Surveys of the adult population have reported a one-year prevalence of 23% for unspecified dizziness and 5% for vestibular vertigo [1]. In comparison, a recent review of all ICD-9 codes related to vestibular and balance disorders in more than 560000 distinct pediatric patient encounters during a 4-year period revealed prevalence of only 0.4% for unspecific dizziness, 0.03% for peripheral, and 0.02% for central vestibulopathy [2].

The first reference in the modern scientific literature for pediatric vertigo was published by Harrison [3] in 1962. Despite the most significant technological achievements in the development of diagnostic tools since then, diagnosis is still based mainly upon the patient's history and physical examination. When a child or an adolescent presents with dizziness, he or she is first being evaluated by the primary physician, usually a pediatrician, and only some are diagnosed with true vertigo. Dizziness and vertigo might present a considerable pathology, and patients are often referred to additional tests or further evaluation performed by either an otolaryngologist or a neurologist [4–6].

The most important clues to the diagnosis of vertigo are obtained through a careful and pertinent clinical history. However, when a child as the patient is considered this task might be hampered due to lack of communication abilities, narrowed vocabulary, and distractibility. These difficulties sometimes lead to the erroneous impression that the presenting symptoms are secondary to lack of coordination or behavioral problems [7]. Due to these limitations meticulous physical examination and laboratory tests are important stepping stones towards the correct diagnosis. Yet, the pediatric patients' compliance may also be limited in the performance of a complete otoneurological evaluation. A further challenge is presented by the remarkable ability of most children to compensate for static vestibular deficits. Vestibular insults that often result in disequilibrium and considerable daily activities limitation in the adult would often show no evidence for such symptoms in the pediatric patient.

The differential diagnosis of childhood vertigo differs from that of adults. Several etiologies are unique to the pediatric population while [5] the occurrences of other pathologies are rather different in children and adults [8, 9].

The aim of this study was to present the etiologies of vertigo in children and adolescents and to describe its course over time.

## 2. Materials and Methods

In a retrospectively designed study we have looked at a cohort of children and adolescents presenting to a tertiary otoneurology clinic with vertigo between the years 2003–2010. The study included patients younger than 18 years with normal otoscopy who had suffered at least one episode of vertigo and had no prior otoneurological evaluation.

Acute otitis media and otitis media with effusion are common causes for unsteadiness or vertigo in children [16]. However, in the present study we focused on children and adolescents presenting to the clinic with vertigo albeit normal otoscopic findings.

We have excluded patients with prior cranial or neurosurgical surgery, previous otoneurological evaluation, or documented developmental disorders.

A detailed medical history of vestibular and migraine symptoms was obtained from the patient and his parents. All patients had a complete otolaryngological and otoneurological physical examination, and audiological evaluation including pure tone, speech, and admittance audiometry. Further tests such as electronystagmography (ENG), auditory brainstem response (ABR), computerized tomography, and magnetic resonance imaging were carried out as indicated.

Follow-up was performed using a telephone-administered questionnaire completed by a parent addressing continuous or recurrent symptoms, need for further evaluation and treatment, overall wellbeing, and compliance with the recommended treatment (See the appendix).

An ethical committee approval was granted for this study.

## 3. Results

A total of thirty-seven patients were included in the study. Patients' demographics and symptoms on presentation are detailed in Table 1.

Bedside examination has documented spontaneous nystagmus in ten (27%) patients, post-head-shaking nystagmus in six (16%), positive head impulse test in five (14%), and positional nystagmus in two (5%).

ENG was indicated in twenty-seven patients. Twenty-three children completed the study successfully while four did not comply with the test. Of the patients completing the ENG sixteen (70%) had pathological caloric test defined as canal paresis >25% or directional preponderance >30% according to Jongkees formula [17]. Other pathological findings included positional nystagmus in two patients, abnormal oculomotor function in one patient, and spontaneous nystagmus in one patient.

Audiological evaluation revealed high tone hearing loss in six patients (16%) and low tone hearing loss in three (8%). One patient had unilateral profound hearing loss.

Thirteen (35%) patients had a brain CT prior to their referral to our clinic. None have revealed findings that contributed to the patient's diagnosis.

Brain MRI was performed in eight (22%) patients and was positive for demyelinative changes in one.

**Figure 1:**
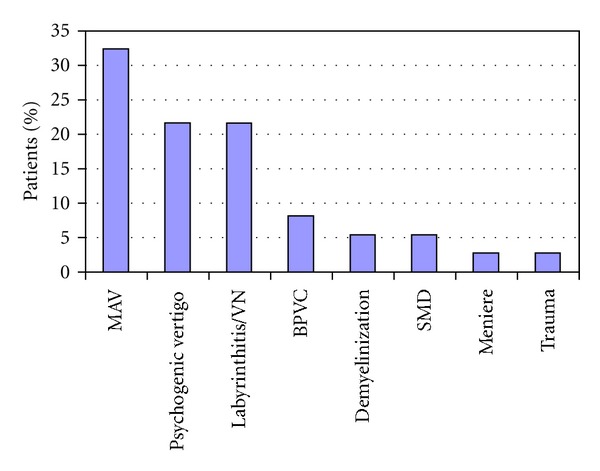
Distribution of the various etiologies for vertigo. MAV: migraine-associated vertigo, VN: vestibular neuritis, BPVC: benign paroxysmal vertigo of childhood, and SMD: space and motion discomfort.

The most common etiology for vertigo was migraine (twelve patients, 32%), followed by acute labyrinthitis/neuritis (eight patients, 22%) and psychogenic dizziness (eight patients, 22%) (Figure 1).

A telephone follow-up questionnaire was completed by twenty six (70%) of the study participants 2–8 years after presentation. Follow-up drop-outs were due to failure in locating the patient in ten cases and refusal to complete the questionnaire in one case. Of the completed questionnaires nine were related to the migraine-associated vertigo (MAV), seven to the labyrinthitis/neuritis, and six to the psychogenic subgroups, respectively. All nine followed-up MAV patients had continuous vertigo complaints, in comparison to three (50%) of the psychogenic dizziness subgroup and two (29%) of the labyrinthitis/neuritis subgroup (Table 2). Four patients, all suffering from MAV, have received pharmacological treatment at the time of follow-up.

Twenty-one patients and/or parents from the twenty six (81%) that were available for follow-up were satisfied with the otoneurological evaluation and management.

## 4. Discussion

Vestibular disorders in the pediatric population assume higher profile in recent years as data and studies highlight its importance in general pediatric healthcare.

The main observation of this study is the diversity of etiologies that might be presented by the leading symptoms of vertigo in the pediatric population. Detailed anamnesis aiming to maximal collaboration of the patient and his caregiver combined with a comprehensive otoneurological bedside examination are essential and most of the time sufficient steps towards the correct diagnosis and recommended treatment approach.

Our experience corroborated by previous publications (Table 3) shows that the etiology of vertigo in a significant percent of patients is migraine. Migrainous headache might accompany the vertigo but often dominate the clinical picture years after the initial presentation. MAV is much more common in the pediatric when compared to the adult population. While MAV was reported in up to 35% of vertigo patients in the pediatric population it was diagnosed in only 6% of adults suffering from vertigo [18]. Recent data demonstrate the potential underdiagnosed extent of MAV in children. While approximately 10% of children meet the International Headache Society criteria for migraine headache, vestibular symptoms occur in about 25% of them [19].

All nine patients suffering from MAV in our cohort that have completed the follow-up questionnaire reported on persistent vestibular symptoms with significant impact on their daily activities. It is not surprising that this subgroup of patients was the least satisfied with the medical treatment they have received. Still, less than half of them used appropriate acute or prophylactic antimigraine medications. This data emphasizes the need for proactive interdisciplinary follow-up of these patients to avoid under-treatment and deterioration of their quality of life.

While vestibular neuritis and labyrinthitis are reported common etiologies for pediatric and adolescents vertigo also in other studies, the incidence of psychogenic vertigo which was found in 22% of our patients is much smaller. Psychogenic dizziness was reported only in three studies with incidence of 5–17% [7, 9, 14], while most reports did not mention it among the etiologies for vertigo in children (Table 3). It is interesting that a recent study focusing on unexplained neurological complaints in children including vertigo, dizziness, headache, and fainting has reported that over 90% of the patients had at least one psychiatric disorder [20].

Benign paroxysmal vertigo of childhood (BPVC) which was found in only three (8%) patients in the present study was a common etiology for childhood vertigo in most other reports (Table 3). BPVC is considered a centrally originated pathology in the spectrum of migraine disease, and some cases develop headaches and present with MAV later in their clinical course [21].

Differences in the study design, inclusion, and exclusion criteria might explain the discrepancies in the reported incidence of various etiologies between the present and previous works. Some of the previous studies have included patients with dizziness or vertigo while other works focused on vertigo alone [6, 9, 10, 12, 15, 22, 23]. The present study included only patients with complaints of whirling vertigo while patients with otoscopic findings of acute or chronic otitis media as well as serous otitis media were excluded. Also, children with previously known neurological deficits were not included in the study.

The value of imaging and neurophysiological laboratory tests should be carefully considered while taking into consideration potential long-term irradiation effects on one hand and the limited compliance of the child to imaging, vestibular, and evoked potential tests that require optimal cooperation on the other hand.

Our experience shows that the value of head CT in the evaluation of the dizzy child is very limited. Head CT results did not contribute to the diagnosis and treatment in any of the 13 patients who were evaluated by this imaging modality. Current standards of risk management for ionizing radiation in the pediatric population [11, 24] combined with the significantly higher sensitivity of brain MRI when posterior fossa and inner ear structures are considered support the use of MRI as the study of choice when brain imaging is indicated [25]. It is important to realize that vertigo due to posterior fossa tumor is uncommon in the pediatric and adolescent population and is found in less than 1% of cases [26]. A retrospective review of 87 children with vertigo who underwent neuroimaging demonstrated new findings in 23 patients. However, 19 of the patients had additional neurological deficits and 3 had intense headaches. The authors concluded that neuroimaging will not aid the diagnostic workup when the only presenting symptoms is vertigo [13].

ENG is an important adjunct in the otoneurologist armamentarium, but compliance with this test might be poor in the pediatric population. A recent report by Szirmai et al. described 145 patients with dizziness or vertigo [9]. Patients work-up was similar to that of our study and included ENG testing. 66% of the study participants completed the ENG test battery compared to 62% in the present study. The ENG findings were pathological in 80% in comparison to 70% in our cohort. Valente [27] in her recent update on vestibular evaluation in the pediatric patient stated that while the underlying causes of vertigo and dizziness might be diagnosed on the basis of patient history and clinical bedside testing, laboratory vestibular tests play an important role in reaching and ascertaining the final diagnosis.

We believe that ENG can contribute to the diagnostic evaluation especially when the clinical picture is obscure. It should be remembered that although compliance is a problem, collecting reliable information from a child is challenging, and objective tests might assume higher importance in these clinical scenarios. A possible alternative to the ENG is the computerized rotatory chair test [28]. The seated patient is exposed to a series of sinusoidal angular accelerations directly stimulating the horizontal semicircular canals while the resulting vestibule-ocular reflex response is recorded. When compared to the ENG calorics this test employs a significantly less provocative stimulus while accurately recording the vestibular response to multiple graded stimuli which better reflect the vestibular function. The mild nature of the stimuli and the possibility to conduct the test while seated on the parent's laps enables good compliance of the pediatric patient [29]. The high cost of the system is a significant limitation and it is not vastly available for clinical use.

While our patient's work-up protocol on presentation was similar to that of previous studies, follow-up results have not been previously reported. Meticulous follow-up is the only way to get important insights on the natural history of the various etiologies for childhood vertigo and vital information on the success of treatment and patient's compliance with health-providers recommendations. The present retrospective study is limited in the extent of successful follow-up that included only 70% of the study participants. We believe that a large prospective study which would include long-term follow-up examinations is warranted. 

## Figures and Tables

**Table 1:** Patient's characteristics on presentation.

Total number of patients		37
Male/female		19/18
Age (years) at presentation (Mean ± SD)		14 ± 3.3
Age range (years)		6–19

Symptoms at presentation	Headache	19 (51%)
	Vomiting	11 (30%)
	Nausea	19 (51%)
	Hearing loss	8 (22%)
	Tinnitus	6 (16%)

Findings on bedside examination	Spontaneous nystagmus	10 (27%)
	Positive post-head-shaking nystagmus	6 (16%)
	Positive head-impulse test	5 (14%)
	Positional nystagmus	2 (5%)

Pathological ENG findings in the 23 completed tests		16 (70%)

**Table 2:** Follow-up outcome of the three major etiology subgroups.

	Migraine-associated vertigo	Psychogenic vertigo	Vestibular neuritis or labyrinthitis
Number of patients	12	8	8
Completed questionnaire	9/12 (75%)	6/8 (75%)	7/8 (88%)
Ongoing symptoms	9/9 (100%)	3/6 (50%)	2/7 (29%)
Symptoms limit daily activities	6/9 (67%)	1/6 (17%)	1/7 (13%)
Ongoing medical follow-up due to vertigo	4/9 (44%)	1/6 (17%)	0/7 (0%)
Current pharmacological treatment	4/9 (44%)	0/6 (0%)	0/7 (0%)
Satisfied with the medical care provided	6/9 (67%)	6/6 (100%)	7/8 (88%)

**Table 3:** Previous studies on dizziness and vertigo in children and adolescents.

	Number of patients	Most common etiologies	
		Peripheral vestibulopathy	29%
O'Reilly et al., 2011 [10]	132	MAV/BPVC	24%
		Developmental delay	11%

		Labyrinthitis/neuritis	22%
Szirmai, 2010 [9]	145	MAV	19%
		Panic or anxiety disorder	17%

		MAV	25%
Wiener-Vacher, 2008 [26]	>2000	BPVC	20%
		Trauma	10%

		Viral infections	28%
Balatsouras et al., 2007 [23]	54	MAV	20%
		BPVC	17%

		BPVC	21%
Niemensivu et al., 2007 [13]	24	MAV	17%
		Otitis media	17%

		MAV	34%
Erbek et al., 2006 [6]	50	BPVC	12%
		Psychogenic vertigo	10%

		BPVC	19%
	119	MAV	14%
Riina et al., 2005 [14]		Vestibular neuritis	12%
		Otitis media	10%
		Psychogenic vertigo	5%

	55	MAV	31%
Choung et al. 2003 [12]		BPVC	26%
		Trauma	7%

		Otitis media	15%
Bower and Cotton, 1995 [15]	34	BPVC	15%
		MAV	12%

MAV: migraine-associated vertigo.

BPVC: benign paroxysmal vertigo of childhood.

## Appendix

Follow-up Questionnaire:
  Pt. #   Initials   Date (yyyy-mm-dd)
  □ □  □ □ □  □ □ □ □-□ □-□ □
Questions:
  - Is the patient under any medical follow-up regarding dizziness?
    - Yes □   
    - No □ 
    - Remarks 
  - Does the patient currently taking any pharmacological therapy?
    - Yes □   
    - No □ 
    - Remarks 
  - Did the patient take any pharmacological therapy in the past?
    - Yes □   
    - No □ 
    - Remarks 
  - If pharmacological therapy used—was it beneficial?
    - Yes □   
    - No □ 
    - Remarks 
  - Did the patient receive any physiotherapy treatments?
    - Yes □   
    - No □ 
    - Remarks 
  - If physiotherapy was conducted—was it helpful?
    - Yes □   
    - No □ 
    - Remarks 
  - To what extent is the patient satisfied from the treatment as a whole (scale of 0–10)?
  - Does the patient still suffer from vertigo?
    - Yes □   
    - No □ 
    - Remarks 
  - If yes; does it interfere with his daily activities?
    - Yes □   
    - No □ 
    - Remarks
